# F-actin dampens NLRP3 inflammasome activity via Flightless-I and LRRFIP2

**DOI:** 10.1038/srep29834

**Published:** 2016-07-19

**Authors:** Danielle Burger, Céline Fickentscher, Philippe de Moerloose, Karim J. Brandt

**Affiliations:** 1 Division of Immunology and Allergy, Inflammation and Allergy Research Group, Hans Wilsdorf Laboratory, Department of Internal Medicine, Faculty of Medicine, University of Geneva, Geneva, Switzerland; 2 Division of Angiology and Hemostasis, University Hospital of Geneva and Faculty of Medicine, Geneva, Switzerland

## Abstract

NLRP3 and ASC are able to form a large multimeric complex called inflammasome in response to a number danger signals. The NLRP3 inflammasome is required for the activation of caspase-1 and subsequent maturation of pro-IL-1β into active IL-1β. Although the mechanisms regulating the formation and activity of NLRP3 inflammasome are yet not fully elucidated, data suggest that the assembly of NLRP3 inflammasome requires microtubules to induce the proximity of ASC and NLRP3. In this study we show that microfilaments (F-actin) inhibit NLRP3 inflammasome activity and interact with NLRP3 and ASC. We demonstrate that the inhibition depends on the actin polymerization state but not on the active polymerization process. In ATP- or nigericin-activated macrophages, our data further indicate that Flightless-I (FliI) and leucine-rich repeat FliI-interaction protein 2 (LRRFIP2) are required for the co-localization of NLRP3, ASC and F-actin. We also established that the ability of Ca^2+^ to accentuate the activity of NLRP3 inflammasome is abrogated in FliI- and LRRFIP2-knockdown macrophages, suggesting that Ca^2+^ signaling requires the presence of FliI and LRRFIP2. Accordingly, we observed that Ca^2+^/FliI-dependent severing of F-actin suppresses F-actin/FliI/LRRFIP2-dependent NLRP3 inflammasome inhibition leading to increase IL-1β production. Altogether, our results unveil a new function of F-actin in the regulation of NLRP3 inflammasome activity strengthening the importance of cytoskeleton in the regulation of inflammation.

Inflammation fulfills important functions in host protection. It participates to pathogen elimination and to the restoration of tissue homeostasis after injury. Uncontrolled inflammation may however result in tissue damage and chronic inflammation. Interleukin-1β (IL-1β) is a key mediator of the inflammatory response^1^,^2^,^3^,^4^. A major regulatory process of IL-1β maturation occurs within inflammasomes^5^,^6^,^7^. Inflammasomes are large multimeric complexes that activate caspase-1, a cystein protease responsible for the processing and subsequent secretion of IL-1β and IL-18, a closely related IL-1 family member^8^,^9^,^10^. A number of nucleotide oligomerization domain (NOD)-like receptor (NLR) family members including NLRP3 have been shown to form inflammasomes in response to various stimuli. The NLRP3 inflammasome consists of the NLRP3 protein, the adaptor protein apoptotic speck-like protein containing a caspase recruitment domain (ASC), and caspase-1. While the mechanisms of NLRP3 inflammasome activation elicited by a wide range of specific agonists including ATP, monosodium urate (MSU) crystals or nigericin, are well documented^5^, the function of Ca^2+^ and the specific molecular mechanism involved in the negative regulation of NLRP3 inflammasome remain to be further defined.

A growing number of studies highlight the emerging importance of cytoskeleton in the regulation of inflammatory responses^11^,^12^,^13^,^14^,^15^,^16^. The cytoskeleton is a dynamic structure constituted of three types of protein filaments: actin microfilaments, microtubules and a group of polymers known collectively as intermediate filaments^17^. Microtubules play a central role in the assembly of NLRP3 inflammasome by promoting the bimolecular interaction between ASC and NLRP3 in response to NLRP3 inflammasome inducers^13^. Actin, a globular protein (G-actin) that polymerizes into long right-handed helix microfilaments called filamentous actin (F-actin), plays an important role in regulating cellular processes such as motility, cytokinesis, and vesicular trafficking^18^. Among proteins known to interact with G- and/or F-actin actin, Flightless-1 (FliI), a member of the gelsolin superfamily of Ca^2+^-dependent actin-remodeling proteins, was shown to serve as a pseudosubstrate and inhibitor of caspase-1^19^. Further data indicate that FliI is recruited to the NLRP3 inflammasome by leucine-rich repeat FliI-interaction protein 2 (LRRFIP2), an NLRP3-associated protein. In this study, we establish that F-actin acts as a negative regulator of NLRP3 inflammasome. We demonstrate that active NLRP3 inflammasome complex co-localizes and interacts with actin microfilaments and that this co-localization requires the presence of FliI and LRRFIP2. Besides controlling the intracellular co-localization of the NLRP3 inflammasome and F**-**actin, we further showed that FliI and LRRFIP2 repress caspase-1 activation, which could be restored through the severing of actin filaments in a Ca^2+^-dependent process.

## Results

### Activated NLRP3 inflammasome interacts with F-actin

**Figure 1:**
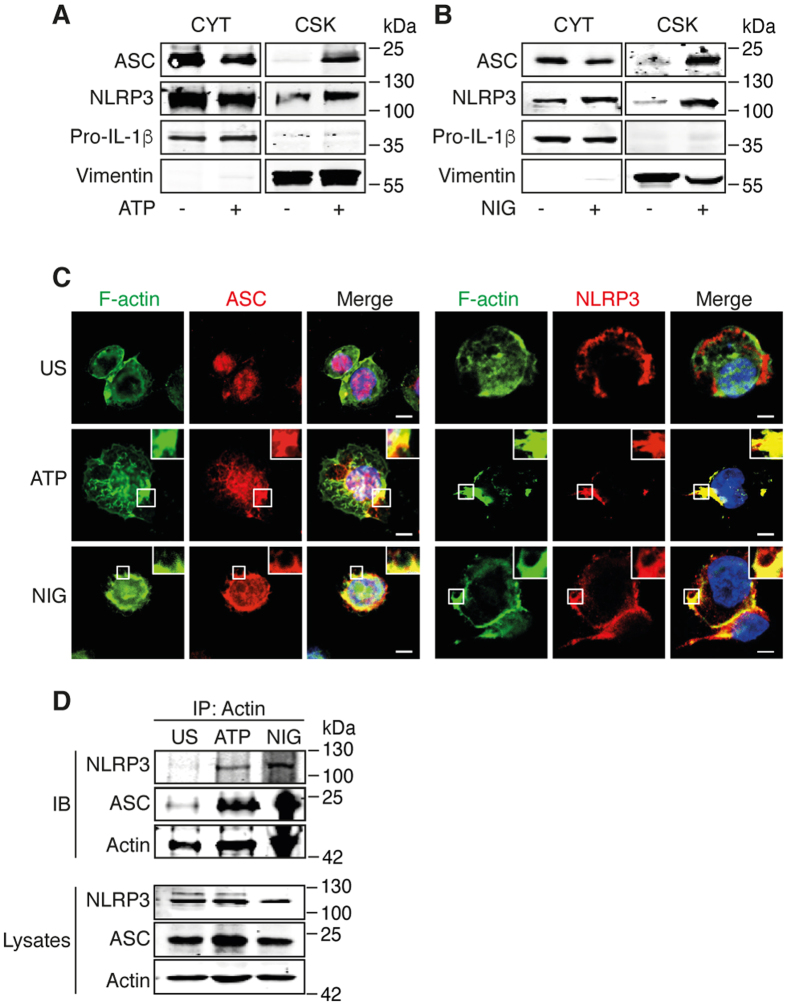
NLRP3 inflammasome interacts with F-actin in ATP- and nigericin-treated THP-1 cells. (**A,B**) Primed THP-1 cells were activated with 5 mM ATP or 1 μM nigericin (NIG) for 6 h. Cytosolic (CYT) and cytoskeletal (CSK) fractions of (**A**) ATP- and (**B**) nigericin-activated cells were subjected to Western blot and analyzed for the presence of ASC, NLRP3, pro-IL-1β and vimentin (control for cytoskeletal fraction). The cropped blots were run under the same experimental conditions; Data are representative of 3 independent experiments. (**C**) Confocal microscopy of ATP- and nigericin-activated THP-1 cells; nuclei are stained in blue. Outlined areas are enlarged in top right corners. Scale bars, 10 μm. Data are representative of 3 independent experiments. (**D**) Primed THP-1 cells were activated with 5 mM ATP or 1 μM nigericin (NIG) for 6 h, immunoprecipitated with anti-actin and subjected to Western blot and analyzed for the presence of ASC, NLRP3 and actin. The cropped blots were run under the same experimental conditions; Data are representative of 2 independent experiments.

To assess the interaction of NLRP3 with the cytoskeleton, we first determined the presence of individual components of the inflammasome into the cytoskeletal or cytosolic fraction of human THP-1 macrophages. While ASC, NLRP3 and pro-IL-1β were detected in the cytosolic fraction of THP-1 cells under steady-state conditions, THP-1 treatment with the inflammasome activators ATP or nigericin induced the translocation of ASC and NLRP3, but not of pro-IL-1β, into the cytoskeletal fraction (Fig. 1A,B), suggesting that the activation of NLRP3 inflammasome triggers its interaction with the cytoskeleton. This observation was further supported by the subcellular co-localization of ASC and NLRP3 with F-actin in ATP- or nigericin-treated THP-1 cells (Fig. 1C). In resting THP-1 cells, ASC was located in the nucleus and the cytoplasm, whereas NLRP3 was only found in the cytoplasm (Fig. 1C). We then addressed whether NLRP3 and ASC could interact with Actin. Co-immunoprecipitation assay revealed that NLRP3 and ASC co-precipitated with actin following stimulation with either ATP or nigericin (Fig. 1D). These results indicate that the activation of NLRP3 inflammasome in THP-1 cells by ATP and nigericin initiates the interaction of NLRP3 and ASC with F-actin.

### F-actin is required for the negative regulation of NLRP3 inflammasome activity

**Figure 2:**
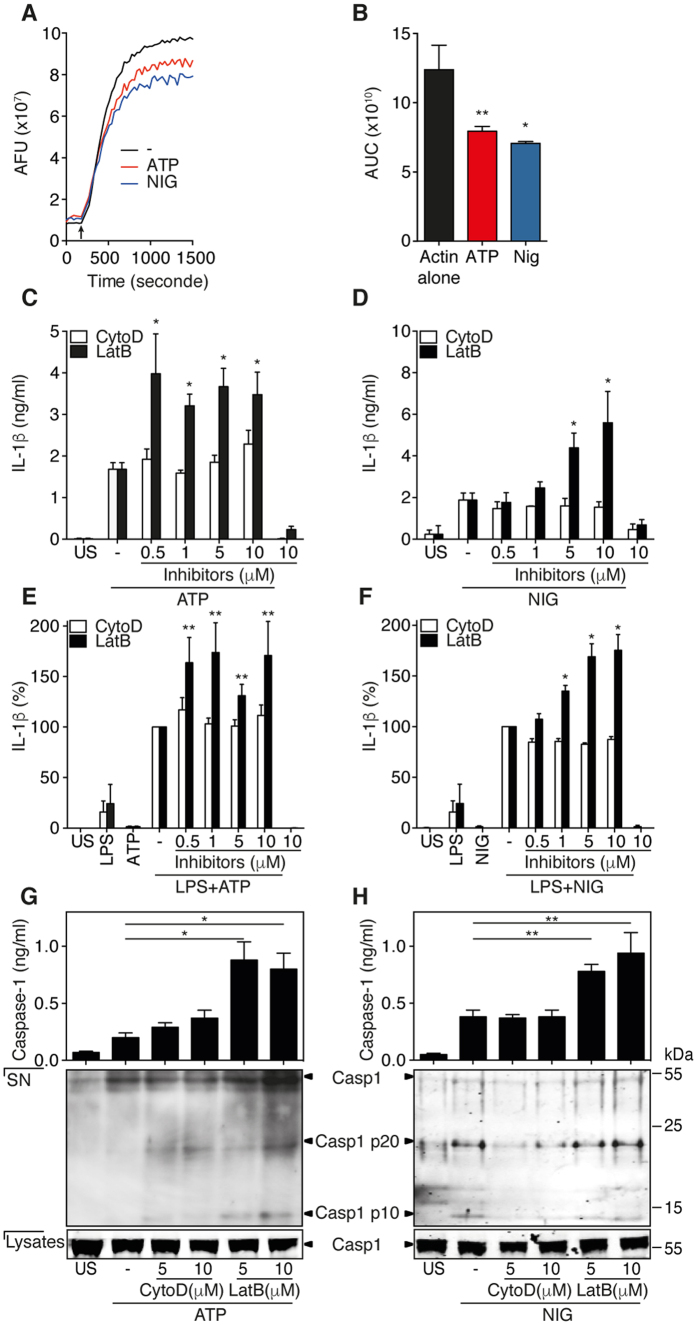
F-actin downregulates NLRP3 inflammasome activity. (**A**) Actin polymerization was performed with 9.6 μM of pyrene-labeled G-actin containing ATP- or nigericin-activated THP-1 cells lysate. The polymerization was initiated by addition of actin polymerization buffer (arrow). Representative experiments out of 3 are presented. (**B**) Area under the curve (AUC) represented in (**A**) was calculated using GraphPad Prism version 6. Data are means ± SEM of at least 3 independent. (**C,D**) IL-1β production in culture supernatants of primed THP-1 cells pretreated with increasing doses of cytochalasin D (CytoD) or latrunculin B (LatB) and then activated by ATP (**C**) and nigericin (**D**) for 6 h. Data are means ± SEM of at least 3 independent. (**E,F**) IL-1β production in culture supernatants of LPS-primed primary human monocytes pretreated with increasing doses of cytochalasin D (CytoD) or latrunculin B (LatB) and stimulated or not with ATP (**E**) or nigericin (**F**) for 15 min. Data are means ± SEM of at least 3 independent. (**G,H**) Caspase-1 in supernatants (SN) of THP-1 cells treated as indicated and assessed by ELISA (upper panels) and Western blot (bottom panels); total, uncleaved caspase-1 in cell lysate is presented in bottom panels. The cropped blots were run under the same experimental conditions. The asterisk indicates nonspecific crossreactive bands. Data are means ± SEM of at least 4 independent experiments (upper panels) or representative of 3 independent experiments (bottom panels). Statistical significance was determined by Mann-Whitney U analysis. See also Figures S1 and S2.

We next assessed whether the interaction of NLRP3 inflammasome with F-actin could affect the dynamic of actin polymerization. To this end, we tested ATP- or nigericin-activated THP-1 cell extracts in a pyrene actin-based assembly assay. As shown in Fig. 2A, these cell extracts potently reduced F-actin assembly after induction of actin polymerization (Fig. 2A, arrow). Concurrently, the areas under the curve (AUC) were significantly diminished by ATP- or nigericin-activated cell extracts (Fig. 2B). We then further evaluate the role of actin dynamics on NLRP3 inflammasome activity using two different cytoskeletal drugs that interact with actin. Latrunculin B, which severs F-actin in addition to sequestering G-actin^20^, significantly decreased the amount of F-actin (Figure S1A,B) and increased IL-1β production in both ATP- and nigericin-treated cells (Fig. 2C,D). As expected cytochalasin D, which sequesters G-actin hampering further actin polymerization^20^, did not affect the G/F-actin ratio (Figure S1A,B) or the production of IL-1β (Fig. 2C,D). Strikingly, the production of IL-18, which also depends on NLRP3 inflammasome, was increased in the presence of latrunculin B but not of cytochalasin D (Figure S1C,D), demonstrating that an active actin polymerization process is not required to inhibit NLRP3 inflammasome activity. Cell viability of treated THP-1 cells was not significantly affected by either cytochalasin D or latrunculin B (Figure S1E,F). We further observed that ZVAD-fmk, an inhibitor of caspase-1, strongly decreased the secretion of IL-1β (Figure S1G,H) and caspase-1 (Figure S1I,J), ruling out the possible release of pro-IL-1β or pro-caspase-1 by dying cells. To ensure that lack of cytochalasin D action on inflammasome activation could not be attributed to a defective biological activity of this compound, its effect was assessed on IL-1β production following phagocytosis of monosodium urate (MSU) crystals^21^, a mechanism that requires active actin polymerization^22^. As shown in Figure S1K, cytochalasin D efficiently inhibited IL-1β production induced by ingestion of MSU crystals. When F-actin was stabilized by jasplakinolide, G-actin could not be detected (Figure S1A,B) and IL-1β production was inhibited (Figure S1L). These results further indicate that F-actin plays an important role in the control of NLRP3 inflammasome-derived IL-1β production. We further found that the NLRC4 activator Flagellin-induced or AIM2 activator Poly(dA:dT)-induced production of caspase-1 and IL-1β were also negatively regulated by F-actin (Figure S1M,N). Importantly, we established that, latrunculin B but not cytochalasin D increased the production of IL-1β (Fig. 2E,F) and IL-18 (Figure S1O) in primary isolated human monocytes primed with LPS and subsequently treated with ATP or nigericin in a same manner than in THP-1 cells. These data indicate that the mechanism by which NLRP3 inflammasome activity is controlled by F-actin is common to both primary monocyte/macrophages and the monocytic cell line THP-1. To further evaluate the ability of F-actin to affect NLRP3 inflammasome-dependent IL-1β maturation and secretion processes, we tested the effects of the various cytoskeletal drugs on the steady state levels of IL-1β mRNA. We found that IL-1β transcript levels remained unchanged upon latrunculin B or cytochalasin D treatment (Figure S2A,B), suggesting that F-actin inhibited only inflammasomes-dependent IL-1β maturation and secretion processes. These data were further strengthened by the observation that activated-caspase-1 levels in cell culture supernatants of THP-1 cells treated with latrunculin B were increased while no significant effects were found in the presence of cytochalasin D (Fig. 2G,H). These data demonstrate that the activation of NLRP3 inflammasomes modulates the amount of F-actin, which is required for the negative regulation of caspase-1 activity and IL-1β secretion.

### Subcellular localization of NLRP3 inflammasome requires F-actin but not active polymerization

**Figure 3:**
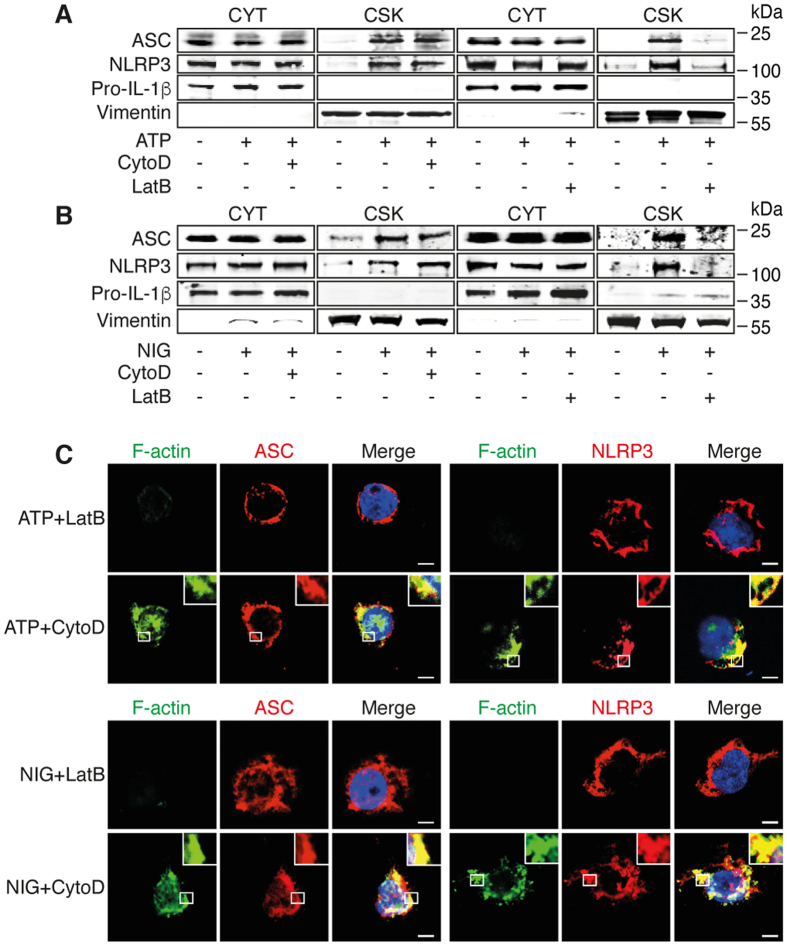
Subcellular location of NLRP3 inflammasome requires F-actin but not active polymerization. (**A,B**) Primed THP-1 cells were activated with (**A**) ATP and (**B**) nigericin after pretreatment with cytochalasin D (CytoD) or latrunculin B (LatB). Cytosolic (CYT) and cytoskeletal (CSK) fractions were analyzed by Western blot. The cropped blots were run under the same experimental conditions; Data are representative of 3 independent experiments. (**C**) Confocal microscopy of subcellular location of ASC and NLRP3 in primed THP-1 cells treated or not with cytochalasin D (CytoD) and latrunculin B (LatB) prior to activation with ATP or nigericin for 6 h; Blue, nuclei. Outlined areas are enlarged in top right corners. Scale bars, 10 μm. Representative pictures of 3 independent experiments.

We then determined the localization of NLRP3 inflammasome components in cytochalasin D- and latrunculin B-treated THP-1 cells after activation with ATP and nigericin. In contrast to cytochalasin D, latrunculin B considerably decreased the amount of NLRP3 and ASC in the cytoskeletal fraction (Fig. 3A,B). Consistent with these observations, the co-localizations of F-actin with NLRP3 and ASC were not affected by the treatment with cytochalasin D but were abrogated in latrunculin B-treated cells (Fig. 3C). These data suggest that the co-localization of the NLRP3 inflammasome and F-actin depends on the actin polymerization state but not on the active polymerization process.

### Inhibition of NLRP3 inflammasome activity by F-actin requires FliI and LRRFIP2

**Figure 4:**
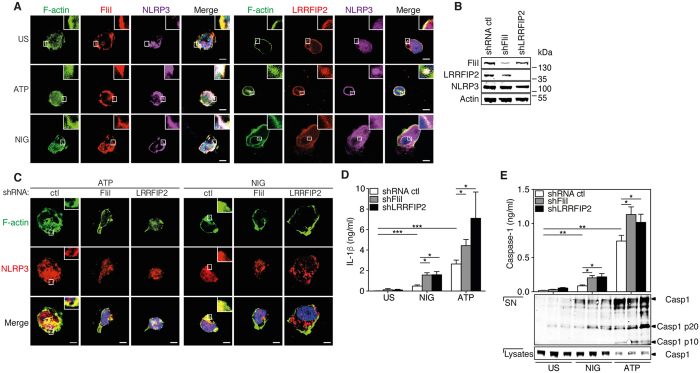
FliI and LRRFIP2 enable inhibition of NLRP3 inflammasome activity via co-localization with F-actin. (**A**) Confocal microscopy of ATP- and nigericin-activated THP-1 cells. Blue, nuclei. Outlined areas are enlarged in top right corners. Scale bars, 10 μm. Representative pictures of 3 independent experiments. (**B**) Western blot analysis of FliI and LRRFIP2 expression in THP-1 cells stably transduced with lentivirus carrying FliI and LRRFIP2 shRNA. Data are representative of 3 independent experiments. (**C**) Confocal microscopy of ATP- and nigericin-activated THP-1 cells transduced with FliI and LRRFIP2 shRNA. Blue, nuclei. Outlined areas are enlarged in top right corners. Scale bars, 10 μm. Representative pictures of 3 independent experiments. (**D**) IL-1β production in culture supernatants of THP-1 cells transduced with FliI and LRRFIP2 shRNA and unstimulated or stimulated with ATP and nigericin. Data are means ± SEM of at least 3 independent. (**E**) Caspase-1 in supernatants (SN) of THP-1 cells treated as indicated and assessed by ELISA (upper panels) and Western blot (bottom panels); the presence of caspase-1 was assessed by Western blot into cells lysate. Data are represented as mean ± SEM of at least 3 independent experiments or representative of 3 independent experiments. Statistical significance was determined by Mann-Whitney U analysis. The cropped blots were run under the same experimental conditions. See Figure S3.

It was previously demonstrated that NLRP3 inflammasome activity is repressed by LRRFIP2 through its interaction with FliI^23^. To determine a possible role for LRRFIP2 and FliI in the interaction between NLRP3 inflammasome and F-actin, we first assessed their respective cellular distribution. While FliI and LRRFIP2 co-localized with F-actin in resting cells, ATP or nigericin treatment induced the recruitment of NLRP3 (Fig. 4A). These data suggest that LRRFIP2 and FliI, a protein known to interact with F-actin^24^, co-localized with activated NLRP3 inflammasome. To further investigate whether FliI and LRRFIP2 regulated NLRP3 co-localization with F-actin, we stably down regulated FliI and LRRFIP2 in THP-1 cells with specific shRNA (Fig. 4B). Silencing of FliI or LRRFIP2 abrogated the co-localization of NLRP3 with F-actin (Fig. 4C) and enhanced the production levels of IL-1β (Fig. 4D) and IL-18 (Figure S3A) as well as caspase-1 activation (Fig. 4E). The expression of IL-1β transcript is not affected by the silencing of FliI or LRRFIP2 (Figure S3B). Together these results indicate that LRRFIP2 and FliI is required for the co-localization of the NLRP3 and F-actin as well as for the inhibition of NLRP3 inflammasome activity.

### Ca^2+^ required the presence of FliI and LRRFIP2 to enhance NLRP3 inflammasome activity

**Figure 5:**
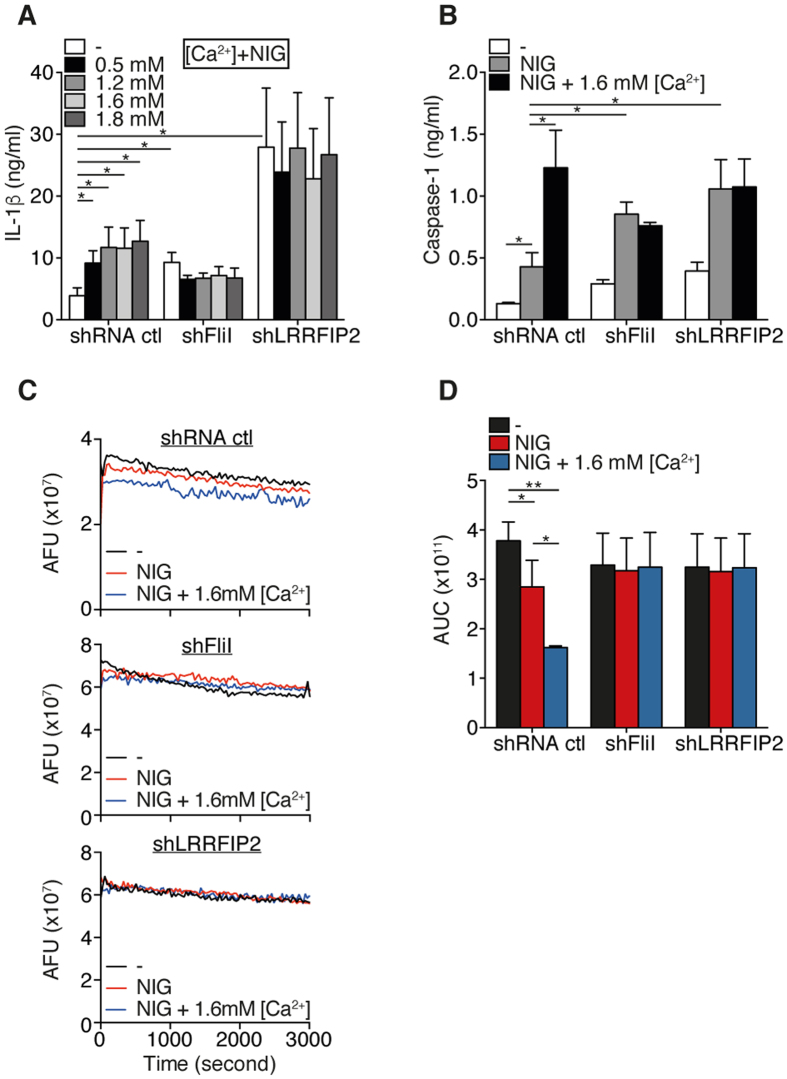
Ca^2+^ increase the NLRP3 inflammasome activation by enhancing the severing of F-actin by FliI. (**A**) IL-1β production in culture supernatants of primed THP-1 cells pretreated with increasing doses of CaCl_2_ (Ca^2+^) and stimulated with nigericin. Data are means ± SEM of at least 3 independent. (**B**) Caspase-1 in supernatants (SN) of THP-1 cells pretreated with 1.6 mM CaCl_2_ and activated by nigericin as indicated and assessed by ELISA. Data are means ± SEM of at least 3 independent. (**C**) Actin depolymerization was performed with 0.2 μg/ml of pyrene-labeled F-actin containing nigericin-activated THP-1 cells lysate. Representative pictures of 3 independent experiments. (**D**) Area under the curve (AUC) represented in (**C**) was calculated using GraphPad Prism version 6. Data are represented as mean ± SEM of at least 3 independent experiments. Statistical significance was determined by Mann-Whitney U analysis. See Figure S4.

FliI belongs to the gelsolin family whose F-actin severing activity depends on Ca^2+ ^25. We thus hypothesized that Ca^2+^ might enhance IL-1β production by promoting actin severing by FliI. As expected, Ca^2+^ enhanced nigericin-induced IL-1β production in a dose-dependent manner in control shRNA transduced-THP-1 cells, whereas it did not affect IL-1β production in FliI- or LRRFIP2-silenced THP-1 cells (Fig. 5A). Importantly, while caspase-1 release induced by nigericin was increased in THP-1 cells independently of FliI or LRRFIP2 silencing, the effects of Ca^2+^ on caspase-1 release were abolished in FliI- or LRRFIP2-silenced THP-1 cells (Fig. 5B). Taken together, these data indicate that Ca^2+^ signaling required the presence of FliI and LRRFIP2 to enhance caspase-1 activation and IL-1β production. To ascertain that Ca^2+^ induced F-actin severing, we carried out a pyrene actin-based depolymerization assay. As expected, while nigericin potently induced F-actin severing, Ca^2+^ enhanced the severing ability of FliI (Fig. 5C). This process was dependent on FliI and LRRFIP2, as determined by the measure of the AUC (Fig. 5D). Interestingly, although LRRFIP2 has no documented severing activity, we observed that extracts derived from cells in which LRRFIP2 had been knocked down displayed the same profile as FliI-shRNA treated cell extract (Fig. 5C,D). While Ca^2+^-dependent effects on NLRP3 inflammasome activity required FliI and LRRFIP2, both proteins were not essential for the inhibitory effects of K^+^ on IL-1β production (Figure S4). These data strongly argue that FliI must interact with NLRP3 to sever F-actin in response to nigericin. The present results unveil a novel mechanism of NLRP3 inflammasome regulation by which intracellular Ca^2+^ enhanced NLRP3 inflammasome activity through the severing of F-actin by FliI ultimately leading to the disruption of the interaction between NLRP3 inflammasome and F-actin.

## Discussion

The present study demonstrates that F-actin acts as a negative regulator of the NLRP3 inflammasome activity. The activation of NLRP3 inflammasome leads to direct interactions of NLRP3 and ASC with F-actin, facilitating the inhibitory effect of FliI on caspase-1 activation and subsequent IL-1β and IL-18 maturation. Besides to the demonstration that both LRRFIP2 and FliI proteins are involved in the regulatory effect of F-actin on caspase-1 activation, our data further indicate that LRRFIP2 and FliI critically control the Ca^2+^ signaling required for the modulation of the NLRP3 inflammasome activity.

Perturbation of actin polymerization by pathogens was recently shown to activate the pyrin inflammasome, a pathogenic mechanism that might be related to the pathogenesis of autoinflammatory diseases that is dependent on IL-18, but not IL-1β^12^. Specifically, the pyrin inflammasome has been reported to be modulated by the Rho GTPases, which have emerged as new regulators of the actin cytoskeleton^26^,^27^. Of note, pyrin and ASC are co-localized to cellular sites that are rich in polymerizing actin^15^. Here we demonstrate that the activation of NLRP3 inflammasome decreases the amount of cellular F-actin and that the negative regulation of the NLRP3 inflammasome by F-actin occurs independently of actin polymerization (Fig. 2). This conclusion is further supported by the observation that cell swelling, a process associated with a decrease of cellular F-actin^28^,^29^, can activate NLRP3 inflammasome^30^. Altogether, these studies suggest that the actin cytoskeleton participates to the regulation of NLRP3 inflammasome activity and that it may also contribute to the regulation of the AIM2 and NLRC4 inflammasomes.

LRRFIP2 was reported to negatively regulate NLRP3 inflammasome activation in macrophages by promoting FliI-mediated caspase-1 inhibition^23^. We show that the negative regulation by F-actin relies on the ability of LRRFIP2 and FliI to interact with NLRP3 (Fig. 4C). FliI is a highly conserved member of the gelsolin superfamily of actin-remodeling proteins that sever F-actin in a Ca^2+^-dependent manner^25^,^31^. We demonstrate here that the activation of NLRP3 inflammasome induces severing of F-actin (Fig. 5C) and that the severing of F-actin is abrogated in FliI- and LRRFIP2-knockdowned THP-1 cells. It has been suggested that the enhancement of cytosolic Ca^2+^ concentrations provides an important mechanism by which stimuli activate the NLRP3 inflammasome^32^. In particular, the activity of Ca^2+^-channels such as the transient receptor potential melastatin 7 (TRPM7) and the transient receptor potential vanilloid 2 (TRPV2) channel has been proposed to regulate the activation of the NLRP3 inflammasome^30^. Of specific importance and in regards to our findings, TRPM7 and TRPV2 were shown to regulate F-actin depolymerization^33^,^34^,^35^, suggesting that these channels could control the activity of the inflammasome through the assembly of F-actin. In this context, recent observations suggest that Ca^2+^ is not a critical second messenger for NLRP3 inflammasome activation, but rather acts as a modulator of its activity^36^. In particular, NLRP3 inflammasome responses to K^+^ efflux could be dissociated from changes in cellular concentrations of Ca^2+ ^36. Consistent with these findings, our results show that regardless the presence or absence of FliI and LRRFIP2, K^+^ reduces IL-1β production (Figure S4A,B) while the lack of FliI and/or LRRFIP2 abolishes the potentiating effects of Ca^2+^ on IL-1β and caspase-1 release without affecting the activation of NLRP3 inflammasome by nigericin (Fig. 5A,B). Taken together, these data further suggest that Ca^2+^ is a secondary messenger required for the fine-tuning of NLRP3 inflammasome activity through FliI-dependent severing of F-actin and a central role of F-actin in the negative regulation of the NLRP3 inflammasome.

**Figure 6:**
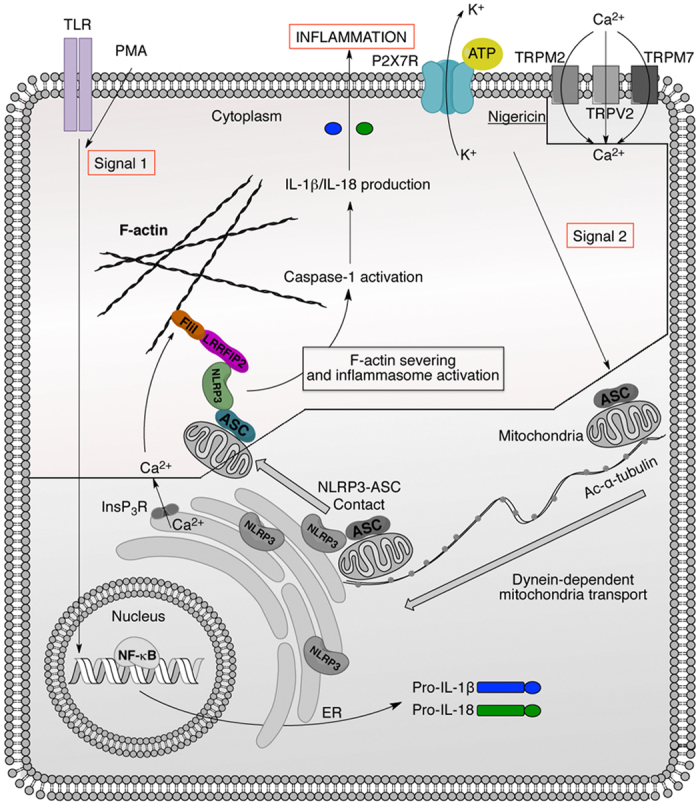
Schematic representation of NLRP3 inflammasome regulation. TLRs activation or PMA (Signal 1) induce the expression of pro-IL-1β, pro-IL-18 and NLRP3. Signal 2 provided by ATP or nigericin induces NLRP3 inflammasome assembly through a dynein- and microtubules-dependent transport. Then, we postulate that the increase of intracellular Ca^2+^ concentration through channels such as TRPM7, TRPV2 and/or InsP_3_R, enhances the ability of FliI to sever F-actin and thus abrogates LRRFIP2-FliI-dependent NLRP3 inflammasome inhibition increasing IL-1β and IL-18 production. The colored part of the picture is the present study.

Complementary to our data, the dynein-dependent transport of mitochondria along microtubules has been found to facilitate the approximation of ASC on mitochondria to NLRP3 on the endoplasmic reticulum in response to NLRP3 inflammasome activators^13^. By considering these different elements together, it is tempting to draw a comprehensive model by which the activity of the NLRP3 inflammasome is regulated. One can envision that increased intracellular concentrations of Ca^2+^ mediated by TRP channels such as TRPM7 and/or TRPV2^30^ enhance the ability of FliI to sever F-actin leading to abrogation of negative regulation of NLRP3 inflammasome activation and maturation of IL-1 family members (Fig. 6). F-actin is used as a docking platform for FliI-LRRFIP2-NLRP3 inflammasome proteins complex leading to negative regulation of caspase-1 and IL-1β production. This model is consistent with most of the currently available experimental evidences. Of specific interest, these various findings also highlight the tight positive and negative regulation in space and time of the NLRP3 inflammasome activity via sequential interactions with microtubules and microfilaments, respectively. This model provides an explanation underlying the role played by Ca^2+^ signaling in the activation of the NLRP3 inflammasome^37^.

In summary, our findings establish that NLRP3 inflammasome activation drives the interactions of NLRP3 and ASC with F-actin as well as regulate the amount of cellular F-actin. We show that both LRRFIP2 and FliI control the localization of NLRP3 with F-actin as well as the activity of NLRP3 inflammasome. Our data further demonstrate that Ca^2+^ signaling, a process requires for modulation of NLRP3 inflammasome activity, is dependent on LRRFIP2 and FliI. Taken together, our results emphasize the complex and interconnected roles of cytoskeleton elements in regulating inflammatory processes.

## Methods

### Ethics statement

Buffy coats of blood of healthy donors were provided by the Geneva Hospital Blood Transfusion Center. All experimental protocols were approved by the ethical committee of the Geneva Hospital and in accordance with the Declaration of Helsinki, the blood bank obtained informed consent from the donors, who are thus informed that part of their blood will be used for research purposes.

### Cells

THP-1 cells (ATCC) were grown in RPMI-1640 medium supplemented with 10% heat-inactivated FCS, 50 μg/ml streptomycin, 50 U/ml penicillin, 2 mM glutamine (medium) and 0.05 mM β-mercaptoethanol. In all experiments, THP-1 cells were primed with 500 nM PMA for 3 h, washed and plated in 48-well plates (1 × 10^6^ cells/0.5 ml/well) for 24 h. Primed THP-1 cells were used as described below. Monocyte purity routinely consisted of >90% CD14+ cells, <1% CD3+ cells and <1% CD19+ cells as assessed by flow cytometry. Cells were cultured in RPMI containing 10% fetal bovine serum (FBS; Gibco BRL-Life Technologies, Zug, Switzerland).

### IL-1β and caspase-1 production

Primed THP-1 cells were treated for 45 min with the indicated inhibitor in Opti-MEM medium (Life Technology) prior to activation by 5 mM ATP, 1 μM nigericin, 500 μg/ml MSU crystals, 5 μg/ml Poly(dA:dT)/LyoVec™ (Invivogen) or 3 μg FLA-PA (transfected with ViaFect) for 6 h (MSU crystals were a kind gift of Dr. A. Scanu, University of Padova). Culture supernatants were tested for the production of IL-1β or caspase-1 by enzyme immunoassay (Human IL-1β, eBioscience, San Diego, CA, USA and Caspase-1, Quantikine; R&D Systems, Minneapolis, MN, USA).

### Immunoprecipitation

Primed THP-1 cells were activated by 5 mM ATP and 1 μM nigericin for 6 h. The cells were collected and lysed at 4 °C in IP buffer containing 0.5% Nonidet-P40, 0.5% Triton X-100, 50 mM HEPES, pH 7.5, 1 mM EDTA, 150 mM NaCl, 10% glycerol, protease inhibitor cocktail, and phosphatase inhibitor cocktail (Roche), followed by centrifugation. The lysates were precleared by incubation with protein G Sepharose 4B beads (Amersham Biosciences) for 2 h at 4 °C, followed by centrifugation. Anti-ASC, anti-NRLP3 and anti-actin (SantaCruz) were incubated with protein G Sepharose 4B beads for 2 h at 4 °C. Precleared lysates were incubated with antibody-coated beads for 24 h at 4 °C. The beads were recovered by centrifugation and washed 3 times with IP buffer, and the immunoprecipitated proteins were eluted in Laemmli sample-loading buffer for subsequent immunoblot analysis.

### *In vitro* pyrene actin-based polymerization assays

Effects of ATP- and nigericin-activated cells extracts on actin polymerization was assessed through the initial rate of fluorescence increase that occurs during pyrene-conjugated G-actin conversion into F-actin by using Actin Polymerization Biochem Kits obtained from Cytoskeleton (Denver, CO). In brief, cells were lysed after ATP or nigericin treatment in 20 mM Tris pH 7.5 containing 20 mM NaCl and 0.5% Triton X-100 (vol/vol) freshly supplemented with protease and phosphatase inhibitor cocktail (Roche). Cellular debris was removed by centrifugations at 20,000 g at 4 °C for 1 h. The supernatant containing the cleared lysate was immediately subjected to actin polymerization assay according to the manufacturer’s instructions. The polymerization is induced by addition of 10× actin polymerization buffer. The kinetics of fluorescence enhancement were monitored in Corning 96-well solid black flat bottom polystyrene microplate using a The SpectraMax^®^ Paradigm^®^ Multi-Mode Microplate Detection (Molecular Devices, Sunnyvale, CA).

### *In vitro* pyrene actin-based severing assays

Effects of ATP- and nigericin-activated cells extracts on steady-state F-actin levels by using Actin Polymerization Biochem Kits (Cytoskeleton, Denver, CO). In brief, cells were lysed after ATP or nigericin treatment in 20 mM Tris-HCl pH 7.5 containing 20 mM NaCl and 0.5% Triton X-100 (vol/vol) freshly supplemented with protease and phosphatase inhibitor cocktails (Roche). Cellular debris was removed by centrifugations at 20,000 g at 4 °C for 1 h. F-actin stock was induces by addition of 10× actin polymerization buffer to Pyrene G-actin solution 1 h prior the addition of the cell lysates according to the manufacturer’s instructions. The severing of pyrene F-actin was revealed by a reduction in the fluorescent signal monitored in Corning 96-well solid black flat bottom polystyrene microplate using a the SpectraMax^®^ Paradigm^®^ Multi-Mode Microplate Reader (Molecular Devices, Sunnyvale, CA).

### mRNA quantification

Primed THP-1 cells were treated with the indicated inhibitors for 45 min in Opti-MEM medium prior to be activated by 5 mM ATP or 1 μM nigericin for 6 h. Total mRNA was prepared by Tri^®^Reagent (Sigma Chemical Co.), according to the provider protocol. Real-time duplex qPCR analysis was conducted as described elsewhere^38^,^39^. The levels of mRNA expression were normalized against the expression of a housekeeping gene (18S) analyzed simultaneously. IL-1β and 18S probes were purchased from Applied Biosystems. All measurements were conducted in triplicate.

### Western blot analysis

Primed THP-1 cells were treated with the indicated inhibitor for 45 min in Opti-MEM prior to be activated by 5 mM ATP or 1 μM nigericin for 6 h. Proteins in cell supernatants^39^ or cell lysates were precipitated and subjected to Western blot analysis as described previously^40^. Membranes were probed with anti-NLRP3 (Cell Signaling, Boston, MA, USA), anti-IL-1β, anti-Vimentin, anti-ASC and anti-caspase-1 p10 and p20 (Santa Cruz Biotechnology, Santa Cruz, CA, USA) and anti-LRRFIP2 (Pierce, Rockford, IL, USA).

### Isolation of cytoplasm and cytoskeleton fractions

Primed THP-1 cells were treated with the indicated inhibitors for 45 min in Opti-MEM prior to be activated by 5 mM ATP or 1 μM nigericin for 6 h. Cell fractionation was performed with ProteoExtract™ Subcellular Proteome Extraction Kit from EMD Millipore Corporation (Billerica, MA, USA). Cell fractions (cytoplasm and cytoskeleton) were subjected to Western blot analysis. Separation of cytoplasm and cytoskeleton was ascertained by assessing the presence of the cytoskeleton protein, vimentin.

### Transduction of shRNA

THP-1 cells were plated in 12-well plates and transduced with control shRNA, FliI and LRRFIP2 lentiviral particles at a MOI equal to 20, according to the supplier’s protocol (Santa Cruz Biotechnology, Santa Cruz, CA, USA). After 72 h, transduced THP-1 cells were selected with puromycin 5 μg/ml.

### Confocal Microscopy

THP-1 cells were primed with 500 nM of PMA for 3 h and plated at 1 × 10^6^ cells/500 μl/well in 8-well Lab-Teck^®^ II Chamber Slide (Nunc, Rochester, NY, USA). After 24 h, THP-1 cells were treated with the indicated inhibitors for 45 min in Opti-MEM prior to be activated by 5 mM ATP or 1 μM nigericin for 6 h. Then, the cells were fixed with 4% paraformaldehyde in PBS for 15 min and permeabilized with 0.1% Triton X100 in PBS for 30 min. Cells were probed with anti-ASC, anti-NLRP3, anti-FliI, and anti-LRRFIP2 (Santa Cruz Biotechnology, Santa Cruz, CA, USA) and phalloidin-FITC (Sigma Chemical Co). The slides were mounted using ProLong^®^ Gold antifade reagent medium with DAPI staining (Life Technologies, USA) and analyzed with an LSM700 Confocal microscope (Zeiss). We used EC Plan Neofluar 63 × 1.4 Oil (Zeiss) for all pictures. The pictures were collected using the Zen 2011 software (Zeiss) and ImageJ (NIH) was used to adjust the contrast and the luminosity.

### Cell Viability

Cells viability was assessed with LDH-Cytotoxicity Assays (BioVision incorporate, Milpitas, CA, USA).

### Statistical analysis

When required significance of differences between groups was assessed using the nonparametric Wilcoxon Mann-Whitney test. *p ≤ 0.05; **p ≤ 0.005; ***p ≤ 0.0005. All data are represented as mean ± SEM of at least 3 independent experiments.

## Additional Information

**How to cite this article**: Burger, D. *et al*. F-actin dampens NLRP3 inflammasome activity via Flightless-I and LRRFIP2. *Sci. Rep*. **6**, 29834; doi: 10.1038/srep29834 (2016).

## Supplementary Material

Supplementary Information
