# Supplementary material for: F-actin dampens NLRP3 inflammasome activity via Flightless-I and LRRFIP2
Source: Sci Rep. 2016 Jul 19;6:29834. doi: 10.1038/srep29834 (PMC4949445; doi:10.1038/srep29834)
Supplement: Supplementary Information [file srep29834-s1.docx]

F-actin dampens NLRP3 inflammasome activity via Flightless-I and LRRFIP2

Danielle Burger^a^, Céline Fickentscher^b^, Philippe de Moerloose^b^, Karim J. Brandt^b^

^a^Division of Immunology and Allergy, Inflammation and Allergy Research Group, Hans Wilsdorf Laboratory, Department of Internal Medicine, Faculty of Medicine, University of Geneva, Geneva, Switzerland; and ^b^Division of Angiology and Hemostasis, University Hospital of Geneva and Faculty of Medicine, Geneva, Switzerland; K.J.B is currently affiliated with Division of Cardiology, Department of Internal Medicine, Faculty of Medicine, University of Geneva, Geneva, Switzerland

# Supplemental information


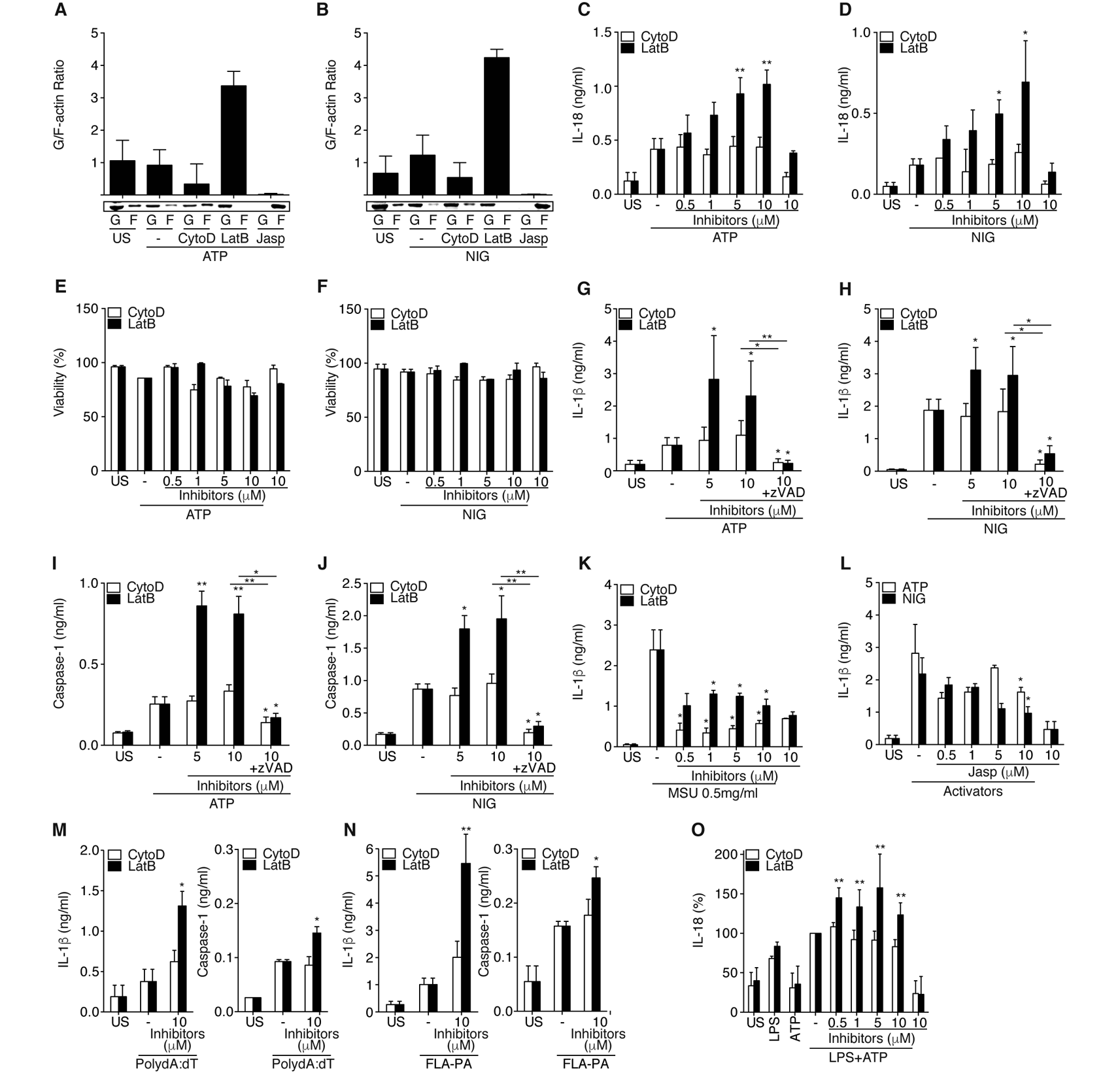


**Figure S1, Related to Figure 2:** (A and B) Latrunculin B but not cytochalasin D severs F-actin. Primed THP-1 cells were activated with (a) ATP or (b) nigericin (NIG) for 6 h in the presence of cytochalasin D (CytoD), latrunculin B (LatB) or jasplakinolide (Jasp) and their content in G and F actin analyzed and quantified by Western blot; data are presented as mean ± SEM of at least 5 independent experiments.

(C and D) IL-18 production in culture supernatants of primed THP-1 cells pretreated with increasing doses of cytochalasin D (CytoD) or latrunculin B (LatB) and then activated by (C) ATP and (D) nigericin for 6h.

(E and F) Viability of primed THP-1 cells upon pre-treatment with increasing doses of cytochalasin D (CytoD) and latrunculin B (LatB) prior to stimulation with (E) ATP and (F) nigericin (NIG).

(G and F) IL-1β production in culture supernatants of primed THP-1 cells pretreated with cytochalasin D (CytoD), latrunculin B (LatB) and a mixture of 20 μM of ZVAD-fmk with 10 μM of indicated inhibitor prior to activation by ATP (G) and nigericin (F) for 6h. Data are represented as mean of percentage ± SEM of at least 3 independent experiments.

(I and J) Caspase-1 secretion in culture supernatants of primed THP-1 cells pretreated with cytochalasin D (CytoD), latrunculin B (LatB) and a mixture of 20 μM of ZVAD-fmk with 10 μM of indicated inhibitor prior to activation by ATP (I) and nigericin (J) for 6h. Data are represented as mean of percentage ± SEM of at least 3 independent experiments.

(K) IL-1β production in primed THP-1 cells pretreated with increasing doses of cytochalasin D (CytoD) or latrunculin B (LatB) prior stimulation or not (US) with MSU crystals. Both cytochalasin D and latrunculin B inhibit the production of IL-1β in MSU crystal-activated THP-1 cells.

(L) IL-1β production by primed THP-1 cells pretreated with increasing doses of jasplakinolide (JASP) prior stimulation or not (US) with ATP or nigericin (activators); Data are represented as mean ± SEM of at least 3 independent experiments.

(M and N) IL-1β and caspase-1 production in primed THP-1 cells pretreated with 10 μM of cytochalasin D (CytoD) or latrunculin B (LatB) prior transfection or not (US) with Poly(dA:dT) or FLA-PA (Flagellin). Data are represented as mean ± SEM of at least 4 independent experiments

(O) IL-18 production in culture supernatants of LPS-primed primary human monocytes pretreated with increasing doses of cytochalasin D (CytoD) or latrunculin B (LatB) and stimulated or not with ATP for 15 min.


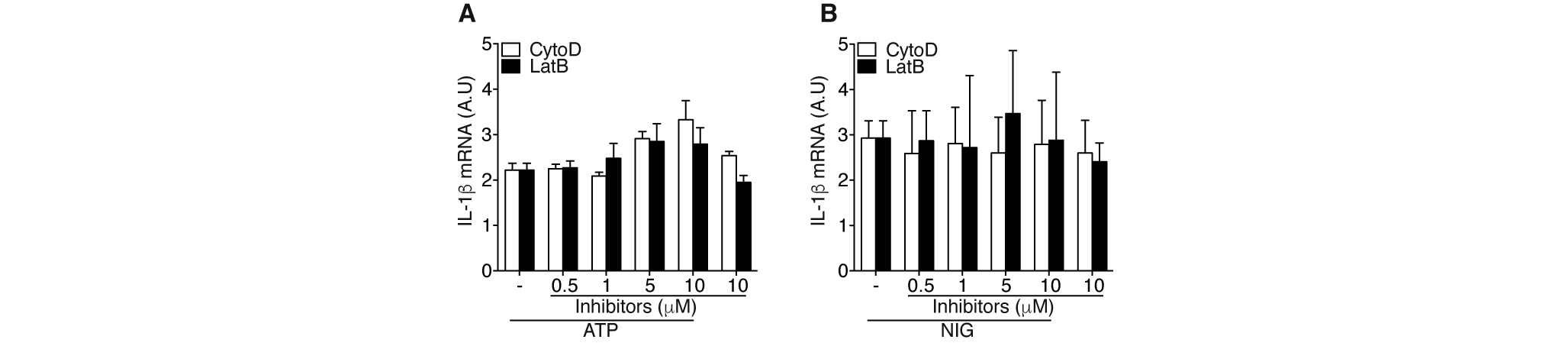


**Figure S2, Related to Figure 2:**

(A and B) F-actin depolymerization does not affect IL-1β transcript levels. Effects of increasing doses of cytochalasin D (CytoD) and latrunculin B (LatB) on IL-1β mRNA expression in primed THP-1 cells unstimulated or stimulated with (a) ATP and (b) nigericin (NIG). Data are presented as mean ± SEM of at least 3 independent experiments.


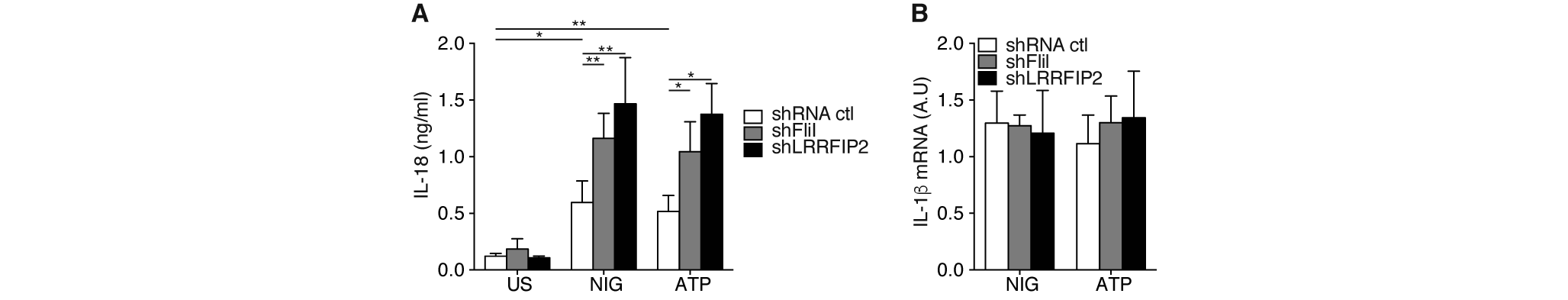


**Figure S3, Related to Figure 4:**

**(A and B)** FliI and LRRFIP2 regulate IL-18 production in ATP- or nigericin-activated THP-1 cells. (A) IL-18 production in culture supernatants of THP-1 cells transduced with FliI and LRRFIP2 shRNA and stimulated or not with ATP and nigericin. (B) FliI and LRRFIP2 do not affect IL-1β mRNA expression. IL-1β mRNA in THP-1 cells transduced with FliI and LRRFIP2 shRNA and unstimulated or stimulated with ATP and nigericin.


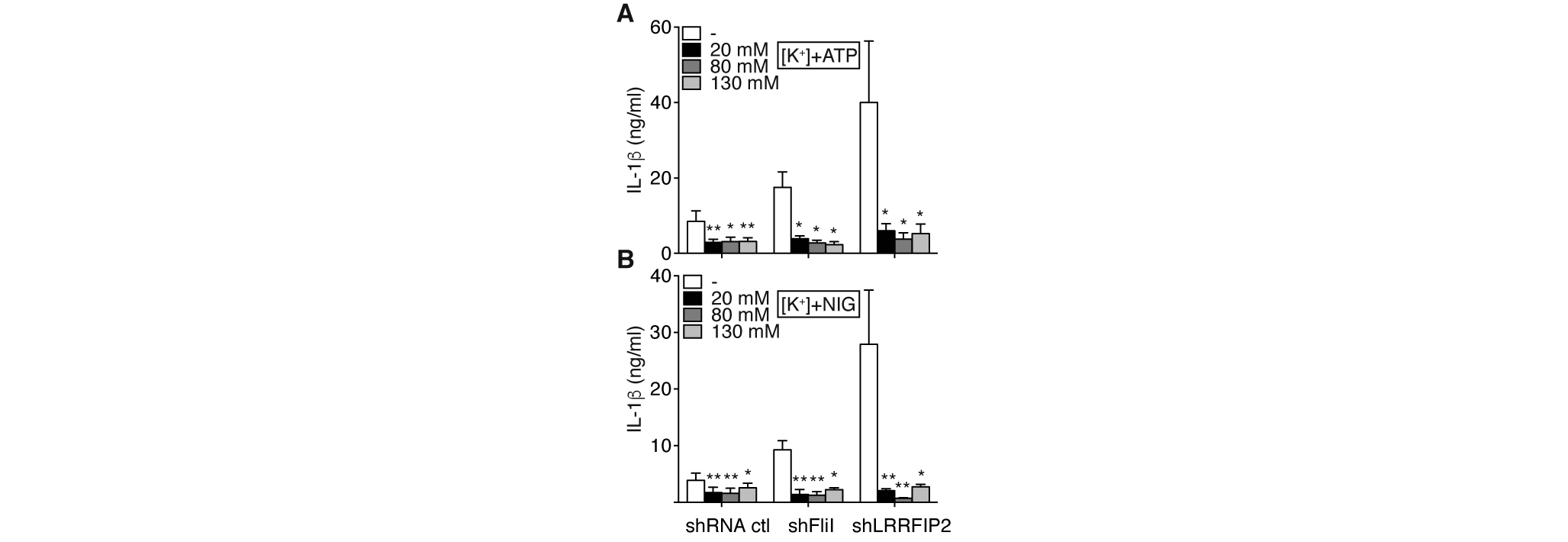


**Figure S4, Related to Figure 5:**

(A and B) K^+^ inhibits the production of IL-1β of nigericin-activated THP-1 cells. IL-1β production in culture supernatants of primed THP-1 cells pretreated with increasing doses of KCl (K^+^) and stimulated with (A) ATP or (B) nigericin.
